# SIRT1 inhibits chemoresistance and cancer stemness of gastric cancer by initiating an AMPK/FOXO3 positive feedback loop

**DOI:** 10.1038/s41419-020-2308-4

**Published:** 2020-02-12

**Authors:** Yifei An, Bo Wang, Xin Wang, Guoying Dong, Jihui Jia, Qing Yang

**Affiliations:** 1 Institute of Pathogen Biology, School of Basic Medical Sciences, Shandong University, Jinan, 250012 China; 2 Department of Traditional Medicine, Qilu Hospital, Shandong University, Jinan, 250012 China; 3 Key Laboratory for Experimental Teratology of the Chinese Ministry of Education, School of Basic Medical Sciences, Shandong University, Jinan, 250012 China; 4 Cancer Research Laboratory, Shandong University, Karolinska Institute Collaborative Laboratory, School of Basic Medical Sciences, Shandong University, Jinan, 250012 China

**Keywords:** Gastric cancer, Gastric cancer, Oncogenesis

## Abstract

Chemotherapy is the standard care for patients with gastric cancer (GC); however, resistance to existing drugs has limited its success. The persistence of cancer stem cells (CSCs) is considered to be responsible for treatment failure. In this study, we demonstrated that SIRT1 expression was significantly downregulated in GC tissues, and that a low SIRT1 expression level indicated a poor prognosis in GC patients. We observed a suppressive role of SIRT1 in chemoresistance of GC both in vitro and in vivo. In addition, we found that SIRT1 eliminated CSC properties of GC cells. Mechanistically, SIRT1 exerted inhibitory activities on chemoresistance and CSC properties through FOXO3 and AMPK. Furthermore, a synergistic effect was revealed between FOXO3 and AMPK. AMPK promoted nuclear translocation of FOXO3 and enhanced its transcriptional activities. In addition, FOXO3 increased the expression level and activation of AMPKα by directly binding to its promoter and activating the transcription of AMPKα. Similar to SIRT1, low expression levels of p-AMPKα and FOXO3a are also related to the poor prognosis of GC patients. Moreover, we revealed a correlation between the expression levels of SIRT1, p-AMPKα, and FOXO3a. These findings indicated the importance of the SIRT1-AMPK/FOXO3 pathway in reversing chemoresistance and CSC properties of GC. Thus, exploring efficient strategies to activate the SIRT1-AMPK/FOXO3 pathway may lead to improving the survival of GC patients.

## Introduction

Gastric cancer (GC) remains the most common cancer worldwide, and is responsible for 1,033,701 new cases, and an estimated 782,685 deaths occurred worldwide in 2018^1^. The high mortality rate is mainly attributed to a late diagnosis and the refractory nature of GC in response to chemotherapy. Despite the recent increase in therapeutic options, combination of 5-fluorouracil (5-FU) and cisplatin remains the generally accepted first-line chemotherapy for GC patients^2^. Due to the development of chemoresistance, the above-mentioned chemotherapy typically fails and thereby promotes GC recurrence in patients^2,3^. Thus, there is an urgent need to make a better understanding of chemoresistance to improve drug responses and develop novel therapeutic strategies.

Tumors consist of heterogeneous cell populations, among which a subpopulation of cells is referred to as tumor-initiating cells. Tumor-initiating cells proliferate, differentiate, and produce all cell types found in a particular tumor; therefore, they are also named cancer stem cells (CSCs)^4^. Compelling evidence has emerged, indicating that the persistence of CSCs is responsible for treatment failure due to the enhanced chemoresistance^4,5^. Moreover, it has been recently reported that CSCs are enriched in response to chemotherapy, which further links CSCs with chemoresistance^6,7^. In addition to the concept of CSC as a defined entity, current data have suggested that CSC is a plastic state, in which epigenetic diversity plays an important role^8,9^. The plasticity of CSCs has motivated efforts to identify epigenetic targets to eliminate cancer stemness and improve chemotherapeutic responses.

Sirtuin 1 (SIRT1) is the founding member of class III histone deacetylases. SIRT1 uses NAD^+^ as a cofactor and the substrates of SIRT1 include histone and nonhistone proteins^10–12^. In addition, dysregulation of SIRT1 has been associated with the pathogenesis of neoplastic, metabolic, infectious, and neurodegenerative diseases^10^. Recent studies have correlated SIRT1 with the function of normal stem cells^13,14^. Nevertheless, the function of SIRT1 in cancer is context dependent. Moreover, the role of SIRT1 in GC chemoresistance, CSC properties, and chemotherapy-induced stemness is largely unknown.

In this study, we demonstrated that downregulated expression of SIRT1 is related to a poor prognosis in GC patients. SIRT1 suppresses chemoresistance and CSC properties of GC through its targets FOXO3 and AMPK. In addition, we also revealed a positive feedback loop between FOXO3 and AMPK. A correlation between SIRT1, p-AMPKα, and FOXO3 was identified using clinical samples.

## Results

### Downregulated expression of SIRT1 is related to a poor prognosis of GC patients

**Fig. 1:**
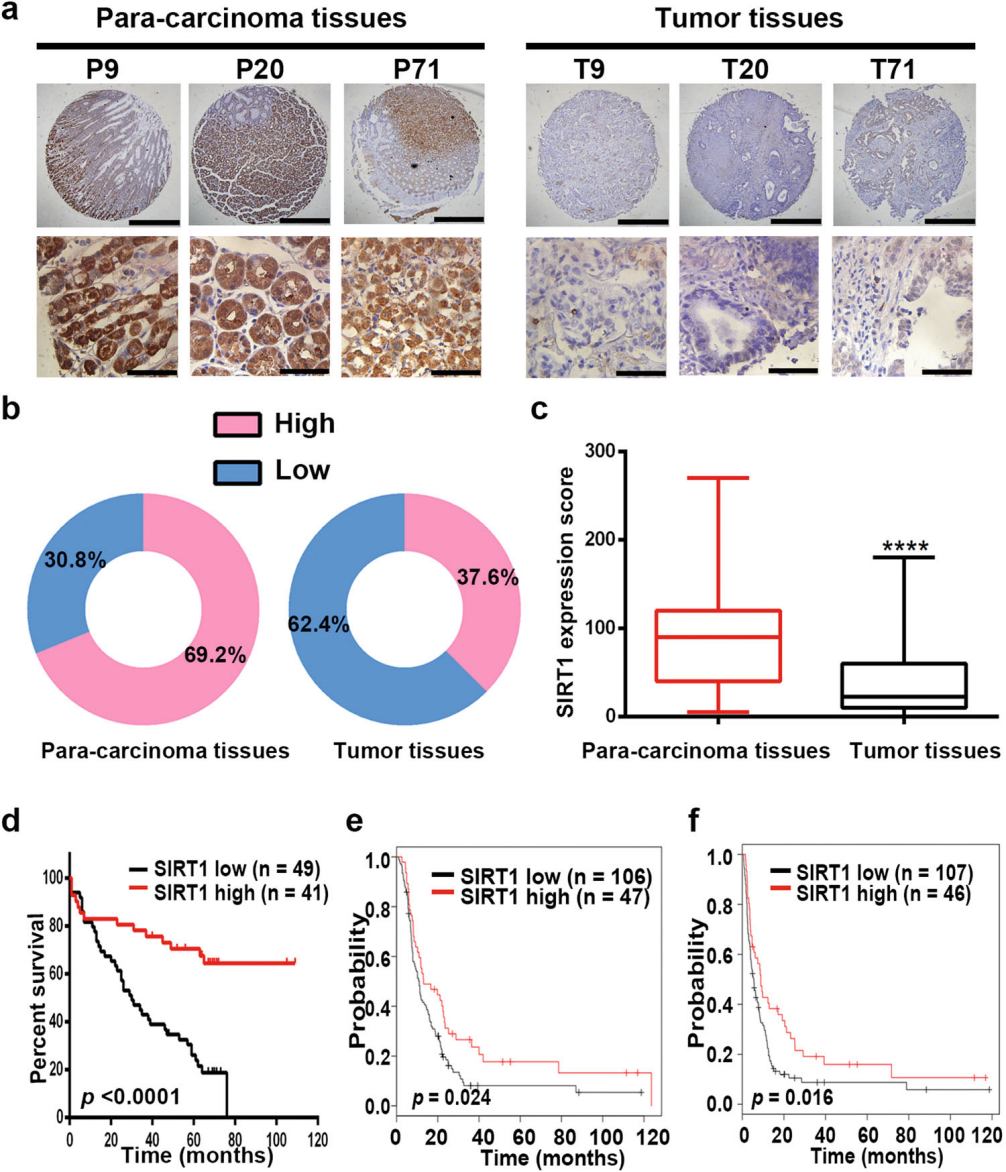
Downregulated expression of SIRT1 is related to a poor prognosis of GC patients. **a** Representative images from human GC (T) and corresponding para-carcinoma (P) tissues stained with SIRT1 (data from the tissue array). Upper panel: ×40, scale bars: 500 µm. Lower panel: ×400, scale bars: 50 µm. **b** SIRT1 expression in human GC (right) and corresponding para-carcinoma (left) tissues (tissue array, *n* = 117). **c** The IHC score (staining intensity × positive percentages) for SIRT1 staining in GC and corresponding para-carcinoma tissues (tissue array, mean ± SD, *n* = 117). **** represents *p* < 0.0001. **d** Analysis of the SIRT1 expression levels in relation to the overall survival of GC patients (tissue array, *n* = 90). **e**, **f** Analysis of the SIRT1 expression levels in relation to the overall survival (**e**) and first progression (**f**) of GC patients treated with a 5-FU-based regimen from the Kaplan–Meier plotter database (218878_s_at) (*n* = 153).

A tissue array was used to examine expression of SIRT1 by IHC staining. The results showed that SIRT1 protein expression was significantly downregulated in GC tissues (Fig. 1a–c). Using univariate Cox regression analysis, we found that depth of tumor infiltration (*p* = 0.03), local lymph node metastasis (*p* < 0.001), clinical stage (pTNM status, *p* = 0.001), tumor grade (*p* = 0.044), and SIRT1 expression levels (*p* < 0.001) were significantly associated with the overall survival of GC patients. Furthermore, multivariate Cox regression analysis further confirmed that local lymph node metastasis (*p* = 0.022) and SIRT1 expression levels (*p* = 0.013) are independent predictors of the overall survival of GC patients (Supplementary Table S1). In addition, high SIRT1 expression levels were associated with good overall survival of GC patients (Fig. 1d). Consistently, the data from Kaplan–Meier plotter database (218878_s_at) also associated higher SIRT1 expression levels with better overall survival and first progression (Supplementary Fig. S1a, b). Moreover, when the analysis was restricted to patients receiving a 5-FU-based treatment, the correlation between higher SIRT1 expression levels and a longer duration of overall survival (Fig. 1e) or a longer period before first progression (Fig. 1f) was significant. The correlation between SIRT1 expression levels and the prognosis of GC patients treated with a 5-FU-based regimen suggests that SIRT1 may be associated with the patient response to chemotherapy.

### SIRT1 inhibits chemoresistance of GC cells

**Fig. 2:**
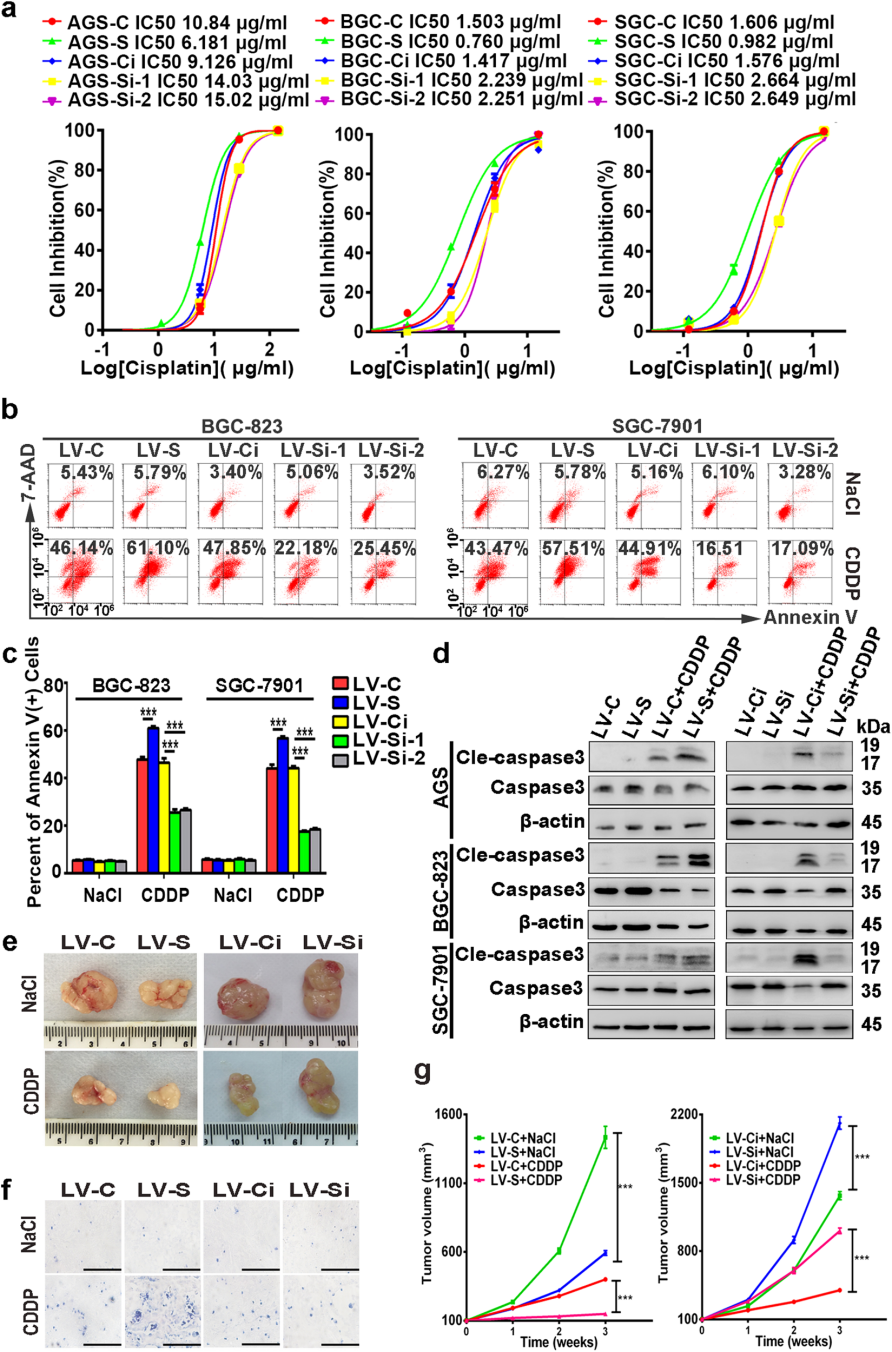
SIRT1 inhibits chemoresistance of GC cells. **a** The IC50 of cisplatin was determined by MTS assays. The mean values of the IC50 are shown (*n* = 3). **b**, **c** The percentages of Annexin V-positive cells upon cisplatin (CDDP) treatment (1.5 µg/ml, 36 h) were examined by flow cytometry. Cells treated with NaCl were used as a control. Data are presented as mean ± SD (*n* = 3). **d** Western blot was used to analyze the expression levels of caspase-3 and cleaved caspase-3 after incubation with cisplatin for 48 h (10 µg/ml for AGS cells and 1.5 µg/ml for BGC-823 and SGC-7901 cells). **e**–**g** Stable SIRT1-overexpression/-knockdown SGC-7901 cells and the corresponding control cells were used for tumorigenesis assays. Cisplatin (5 mg/kg, every 5 days) or an equal volume of NaCl was intraperitoneally injected when the tumor volumes reached 100 mm^3^ (eight mice in each group). After 3 weeks, the mice were euthanized, and the tumor nodules were harvested. Representative images of the tumor nodules (**e**) and TUNEL staining (**f**, ×200, scale bars: 100 µm) are shown. The tumor volumes were measured and shown in (**g**) (mean ± SD, *n* = 8). *** represents *p* < 0.001.

To evaluate the effects of SIRT1 on chemoresistance, stable lentivirus-infected GC cells were used. Cells stably transfected with the lentiviral expression vector of SIRT1 and the control vector were regarded as LV-S and LV-C, respectively. Cells stably transfected with lentiviral shRNA targeting SIRT1 and the negative control were regarded as LV-Si and LV-Ci, respectively. Upon treatment with cisplatin or 5-FU, GC cells overexpressing SIRT1 exhibited enhanced sensitivity. In contrast, silencing of SIRT1 facilitated resistance to cisplatin and 5-FU (Fig. 2a; Supplementary Fig. S2a). To further evaluate cell proliferation after chemotherapy, colony-formation assays were performed. Forced expression of SIRT1 caused a significant reduction in foci numbers and sizes upon cisplatin treatment, while knockdown of SIRT1 caused the opposite effects (Supplementary Fig. S2b, c).

In addition, the effect of cisplatin on cell apoptosis was determined by flow cytometry. Upon cisplatin treatment, higher percentages of apoptotic cells were observed in GC cells overexpressing SIRT1 compared with the controls. In contrast, cells with SIRT1 knockdown showed less apoptosis compared with their controls (Fig. 2b, c). Consistently, the suppressive effect of SIRT1 on apoptosis upon cisplatin treatment was also validated by the protein expression levels of cleaved caspase-3 (Fig. 2d).

Furthermore, we assessed the role of SIRT1 in modulating cisplatin resistance in vivo. Cells with SIRT1 overexpression showed increased sensitivity to cisplatin treatment, as indicated by reduced tumor sizes and increased TUNEL-labeled apoptotic cells. In contrast, cells with SIRT1 silencing showed increased resistance to cisplatin (Fig. 2e–g). Taken together, our results indicated the suppressive role of SIRT1 in chemoresistance of GC cells.

### SIRT1 inhibits CSC properties of GC cells

**Fig. 3:**
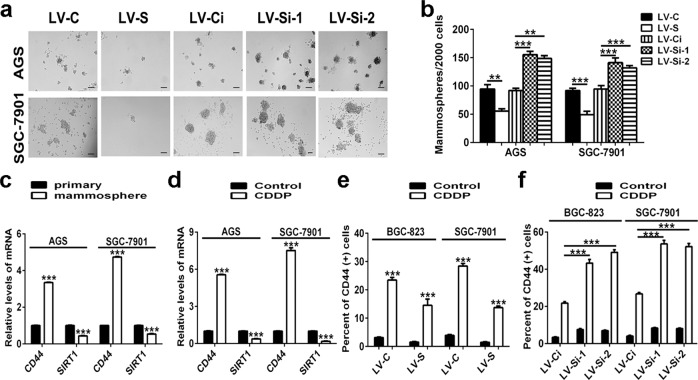
SIRT1 inhibits CSC properties of GC cells. **a**, **b** Mammosphere assays were performed to evaluate the cancer stemness of GC cells. Representative images are shown in (**a**). Data are presented as mean ± SD (*n* = 3). **c**, **d** Real-time PCR was performed to determine the mRNA expression levels of *CD44* and *SIRT1*. The results from GC cells (regarded as primary) and mammospheres obtained from GC cells are shown in (**c**). The results from GC cells treated with cisplatin (CDDP, 10 µg/ml for AGS cells and 1.5 µg/ml for SGC-7901 cells, 48 h) or NaCl (Control) are shown in (**d**). Data are presented as mean ± SD (*n* = 3). **e**, **f** Percentages of CD44-positive cells were detected by flow cytometry. Stable SIRT1-overexpressing (**e**) or SIRT1-knockdown (**f**) GC cells treated with cisplatin (CDDP, 1.5 µg/ml, 48 h) or NaCl (Control) were used. Data are presented as mean ± SD (*n* = 3). ** represents *p* < 0.01, *** represents *p* < 0.001.

Because CSCs are considered to be responsible for chemoresistance, we examined whether SIRT1 is also involved in the maintenance of the CSC phenotype in GC. The results from mammosphere assays demonstrated that overexpression of SIRT1 markedly reduced the spheroid formation abilities of GC cells. Accordingly, SIRT1 knockdown was shown to enhance the spheroid formation abilities of GC cells (Fig. 3a, b). Consistently, the inhibitory role of SIRT1 in CSC phenotype was confirmed by soft agar colony-formation experiments. A significant decrease in foci numbers and sizes was observed in SIRT1-overexpressing GC cells, while silencing of SIRT1 showed the opposite effects (Supplementary Fig. S3a, b). Then, mRNA levels of the classic GC stem cell marker CD44^15^ and levels of important transcription factors for stemness maintenance were analyzed, and were shown to be negatively regulated by SIRT1 (Supplementary Fig. S3c–f, j). Moreover, percentages of CD44-positive cells decreased in GC cells with forced expression of SIRT1, but increased in GC cells with SIRT1 knockdown (Fig. 3e, f). In spheroids (obtained from mammosphere assays), which were considered to be formed by CSCs, the mRNA expression levels of CD44 and the abovementioned transcription factors increased, whereas the mRNA expression levels of SIRT1 decreased (Fig. 3c; Supplementary Fig. S3g). Consistent with the in vitro results, data from in vivo limiting dilution assays showed that mice harboring SIRT1-overexpressing SGC-7901 cells showed impaired tumor-initiating ability, whereas mice harboring SIRT1-knockdown SGC-7901 cells exhibited accelerated tumor formation (Table 1).

**Table 1:** Stem cell frequency of stable lentivirus-infected SGC-7901 cells.

Cell numbers	LV-C vs. LV-S		LV-Ci vs. LV-Si	
	LV-C	LV-S	LV-Ci	LV-Si
2 × 10^6^	5/5	5/5	5/5	5/5
5 × 10^5^	5/5	4/5	5/5	5/5
2 × 10^5^	3/5	0/5	2/5	5/5
5 × 10^4^	0/5	0/5	0/5	3/5
CSC frequency	1/168,666	1/528,150	1/272,391	1/125,574
95% conference interval	1/389,391–1/73,059	1/1,170,808–1/238,248	1/580,997–1/127,711	1/124,101–1/19,011
*p-*value	0.0183		0.0436	

Recently, it has been reported that CSCs are enriched after chemotherapy^6,7^. We examined expression of CD44, transcription factors that are responsible for maintaining stemness, and SIRT1 in GC cells upon cisplatin treatment. The results showed that after cisplatin treatment, the levels of markers for CSCs were upregulated, while SIRT1 expression levels were downregulated (Fig. 3d; Supplementary Fig. S3h, i). Upon treatment with cisplatin, CD44^+^ CSC populations were enriched regardless of SIRT1 expression levels, and were more abundant in GC cells with SIRT1 knockdown. Accordingly, after cisplatin treatment, GC cells overexpressing SIRT1 contained a smaller percentage of CSCs (Fig. 3e, f). Therefore, the above data suggested an inhibitory effect of SIRT1 on CSC properties of GC cells.

### AMPK and FOXO3 serve as targets of SIRT1 and mediate the function of SIRT1 in chemoresistance and CSC properties

**Fig. 4:**
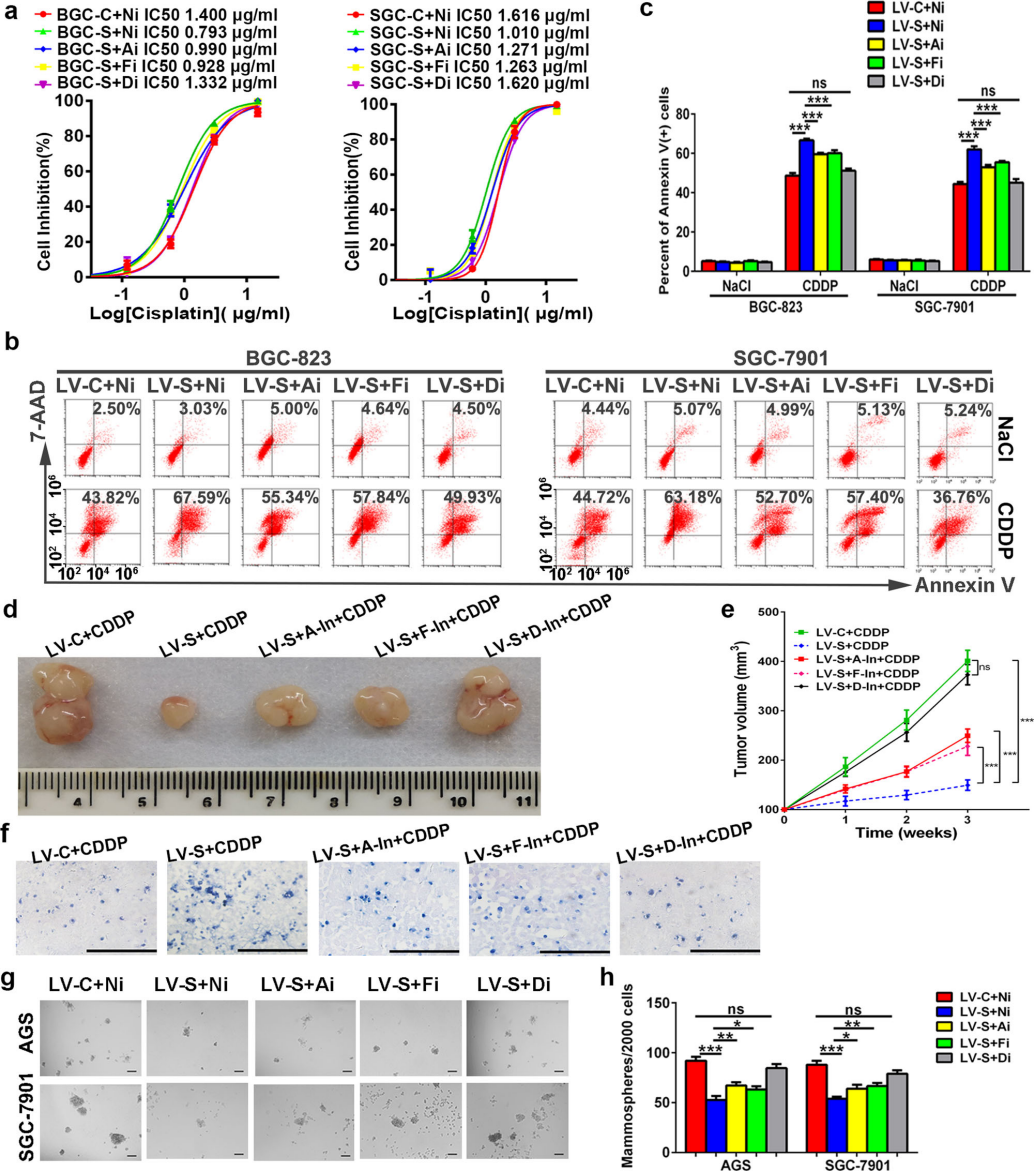
AMPK and FOXO3 mediate the function of SIRT1 in chemoresistance and CSC properties. **a** The IC50 of cisplatin was determined by MTS assays. The mean values of the IC50 are shown (*n* = 3). Ai: small interfering RNA targeting AMPKα, Fi: small interfering RNA targeting FOXO3a, Di: combination of small interfering RNAs targeting AMPKα and FOXO3a, Ni: negative control for small interfering RNA experiments. **b**, **c** Percentages of Annexin V-positive cells upon cisplatin treatment (CDDP, 1.5 µg/ml, 36 h) were examined by flow cytometry. Cells treated with NaCl served as the control. Data are presented as mean ± SD (*n* = 3). **d**–**f** A mouse xenograft model was used to determine the effects of inhibitors of AMPK (Compound C, indicated as A-In), FOXO3 (AS1842856, indicated as F-In) or both of the above two inhibitors (indicated as D-In) in vivo (ten mice in each group). Representative images of tumor nodules are shown in (**d**). The tumor volumes were measured and presented as mean ± SD (**e**). TUNEL staining of xenografts obtained from each group is shown (**f**, ×200, scale bars: 100 µm). **g**, **h** Mammosphere assays were performed to evaluate the cancer stemness of GC cells. Representative images are shown in (**g**). Data are presented as mean ± SD (*n* = 3). * represents *p* < 0.05, ** represents *p* < 0.01, *** represents *p* < 0.001, ns represents not significant.

Next, we investigated targets that are responsible for the inhibitory role of SIRT1 in chemoresistance and CSC properties of GC. STRING database (v11.0) was analyzed to screen for genes that are correlated with SIRT1 and core stemness factors (CD44, OCT4, SOX2, NANOG, and c-MYC). And FOXO3 was identified as a potential functional partner (Supplementary Fig. S4a). In accordance with the data from hematopoietic stem cells^16^, our luciferase assay results indicated that inhibition of SIRT1 suppressed the transcriptional activity of FOXO3 in GC cells (Supplementary Fig. S4b). As expected, knockdown of FOXO3 partially increased spheroid formation in GC cells with forced SIRT1 expression (Fig. 4g, h; Supplementary Fig. S5a, b). This indicates that, in addition to FOXO3, there may be some other targets of SIRT1 that participate in this process. In STRING database, the central metabolic regulator AMP-activated protein kinase (AMPK) was shown to be associated with both SIRT1 and FOXO3 (Supplementary Fig. S4c). Using three different GC cell lines, activation of AMPKα by SIRT1 was validated by examining its phosphorylation after pretreatment with a SIRT1 agonist or anti-agonist (Supplementary Fig. S4d). Then we defined AMPKα substrates of SIRT1 in GC using mammosphere assays. We found that knockdown of AMPKα in SIRT1-overexpressing GC cells partially increased spheroid formation in GC cells with forced SIRT1 expression. However, when we doubly knocked down AMPKα and FOXO3, a complete reversal of inhibited spheroid formation was observed (Fig. 4g, h; Supplementary Fig. S5c, d). This result suggests that both AMPK and FOXO3 serve as targets of SIRT1 in CSC properties of GC and that there may be synergistic effects between these two targets.

For drug response, the results from MTS assays showed that silencing either AMPKα or FOXO3 in SIRT1-overexpressing GC cells partially reversed the chemosensitivity induced by SIRT1. However, when we doubly knocked down AMPKα and FOXO3, a complete reversal of the improved drug response was observed (Fig. 4a). In addition, data from flow cytometry indicated that deletion of either AMPKα or FOXO3 partially decreased the apoptotic cell populations in SIRT1-overexpressing GC cells treated with cisplatin. Nevertheless, the apoptotic percentage of SIRT1-overexpressing GC cells with AMPKα and FOXO3 double knockdown was comparable with that of the control (Fig. 4b, c). In the in vivo tumorigenicity assays, inhibitors were used to suppress the activities of AMPK and/or FOXO3^17^. Consistent with the in vitro findings, suppressing both AMPK and FOXO3 in SIRT1-overexpressing xenograft tumors substantially reversed the improved cisplatin sensitivity, as indicated by the tumor volumes and TUNEL staining (Fig. 4d–f). These findings indicated that both AMPK and FOXO3 are involved in SIRT1 inhibiting chemoresistance and CSC properties of GC cells, and suggested a synergistic effect between these two targets.

### Positive feedback between AMPK and FOXO3

**Fig. 5:**
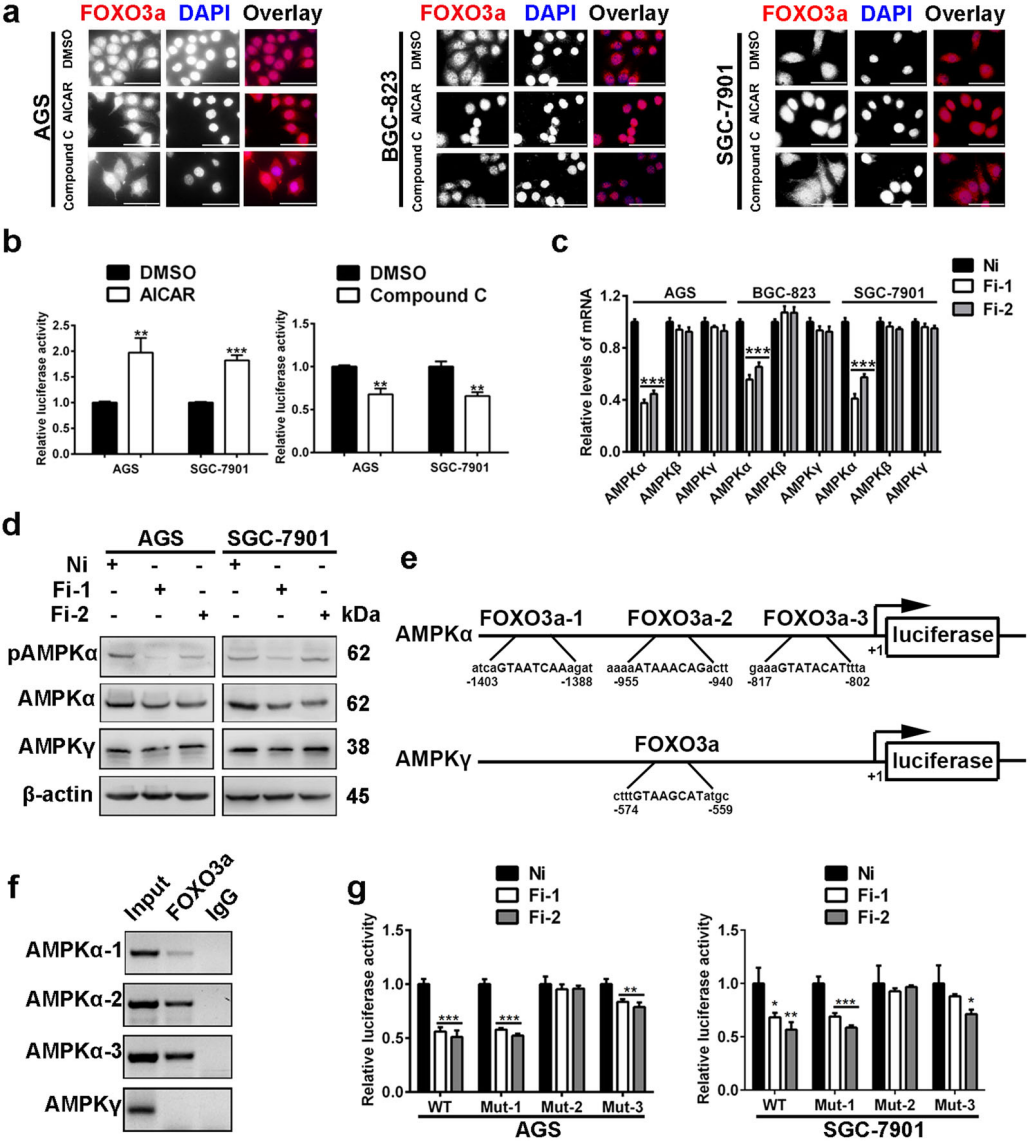
Positive feedback between AMPK and FOXO3. **a** Intracellular distribution of FOXO3a was examined by immunofluorescence staining. The AMPK activator (AICAR, 1 mM, 24 h) promoted nuclear translocation of FOXO3a, while the AMPK inhibitor (Compound C, 10 µM, 24 h) led to cytoplasmic distribution of FOXO3a. Magnification: ×400, scale bars: 50 µm. **b** Transcriptional activity analysis of FOXO3. Cells were pretreated with AICAR (1 mM) or Compound C (10 µM) for 2 h. Data are presented as mean ± SD (*n* = 3). **c** Real-time PCR was performed to determine the mRNA expression levels of the three subunits of *AMPK*. Data are presented as mean ± SD (*n* = 3). Fi: small interfering RNA targeting FOXO3a. Ni represents the negative control. **d** Western blot was performed to analyze the expression levels of AMPKα, p-AMPKα and AMPKγ. **e** The scheme of putative FOXO3-binding sites on the promoters of AMPKα and AMPKγ. **f** ChIP assays showed that FOXO3 directly interacts with the FOXO3-binding sites (mainly the second and the third putative binding sites) within the AMPKα promoter. No binding signal was detected on the AMPKγ promoter. **g** Luciferase activities of different AMPKα promoter constructs in GC cells treated with FOXO3a siRNAs. WT: luciferase reporter vector containing the primary AMPKα promoter, Mut-1, -2, -3: luciferase reporter vector containing the AMPKα promoter with deletion of the FOXO3-binding site 1, 2, 3, respectively. Data are presented as mean ± SD (*n* = 3). * represents *p* < 0.05, ** represents *p* < 0.01, *** represents *p* < 0.001.

Then, we explored whether there is a positive feedback loop between AMPK and FOXO3. For the regulation of FOXO3 by AMPK, we observed subcellular location and transcriptional activities of FOXO3. The results of immunofluorescence staining demonstrated that the AMPK activator enhanced nuclear accumulation of FOXO3a in GC cells. In contrast, the AMPK inhibitor promoted translocation of FOXO3a from the nucleus to the cytoplasm (Fig. 5a). Then, we examined transcriptional activities of FOXO3a. The data from luciferase assays showed increased transcriptional activities of FOXO3a in GC cells treated with the AMPK agonist, while the AMPK inhibitor demonstrated the opposite effects (Fig. 5b).

Next, the role of FOXO3a in AMPK regulation was determined. In nematodes, results reported by Tullet et al.^18^ showed that DAF-16, which is a homolog of mammal FOXO3, directly activates the expression of AMPKγ. However, related studies in mammals have not been performed. Functional AMPK is a heterotrimer consisting of a catalytic α-subunit, a scaffolding β-subunit and a regulatory γ-subunit. We knocked down expression of FOXO3a in GC cells and found that, unlike the condition in nematodes, the mRNA expression levels of AMPKα decreased significantly (Fig. 5c). We also evaluated expression of AMPKα and AMPKγ at protein levels, and the results demonstrated that expression levels of AMPKα, but not AMPKγ, were downregulated by FOXO3a interference. Moreover, phosphorylation of AMPKα was also downregulated by FOXO3a silencing (Fig. 5d). As a transcription factor, FOXO3 has been shown to bind to promoters of target genes and regulate their expression. Therefore, we analyzed promoter sequences of AMPKα and AMPKγ using JASPAR database. Three putative binding sites of FOXO3 were found in the promoter region of AMPKα, and one binding site of FOXO3 was found in the promoter region of AMPKγ (Fig. 5e). Next, we performed ChIP assays to determine the binding of FOXO3 on the promoters of AMPKα and AMPKγ. As shown in Fig. 5f, evident binding signals were detected in the second and third binding sites on the AMPKα promoter. Only a weak band was observed for the first binding site of FOXO3 on the AMPKα promoter. No binding signal was detected for the FOXO3-binding site on the AMPKγ promoter. To further determine whether the binding of FOXO3 on the AMPKα promoter has functional significance, we performed dual luciferase assays. The results revealed that FOXO3 inhibition decreased the luciferase activities driven by the AMPKα promoter. Deletion of the first binding site of FOXO3 did not affect the decrease of luciferase activities. Nevertheless, deletion of the second binding site of FOXO3 almost completely rescued the suppressive role of FOXO3a knockdown. Moreover, deletion of the third binding site of FOXO3 also played a role in the repressive effects of FOXO3 silencing (Fig. 5g). Our results indicated that both the second and the third binding sites of FOXO3 on the AMPKα promoter are necessary for the binding of FOXO3. Taken together, above results uncovered a positive feedback between AMPK and FOXO3.

### Correlation between SIRT1, p-AMPKα, and FOXO3 in clinical samples

**Fig. 6:**
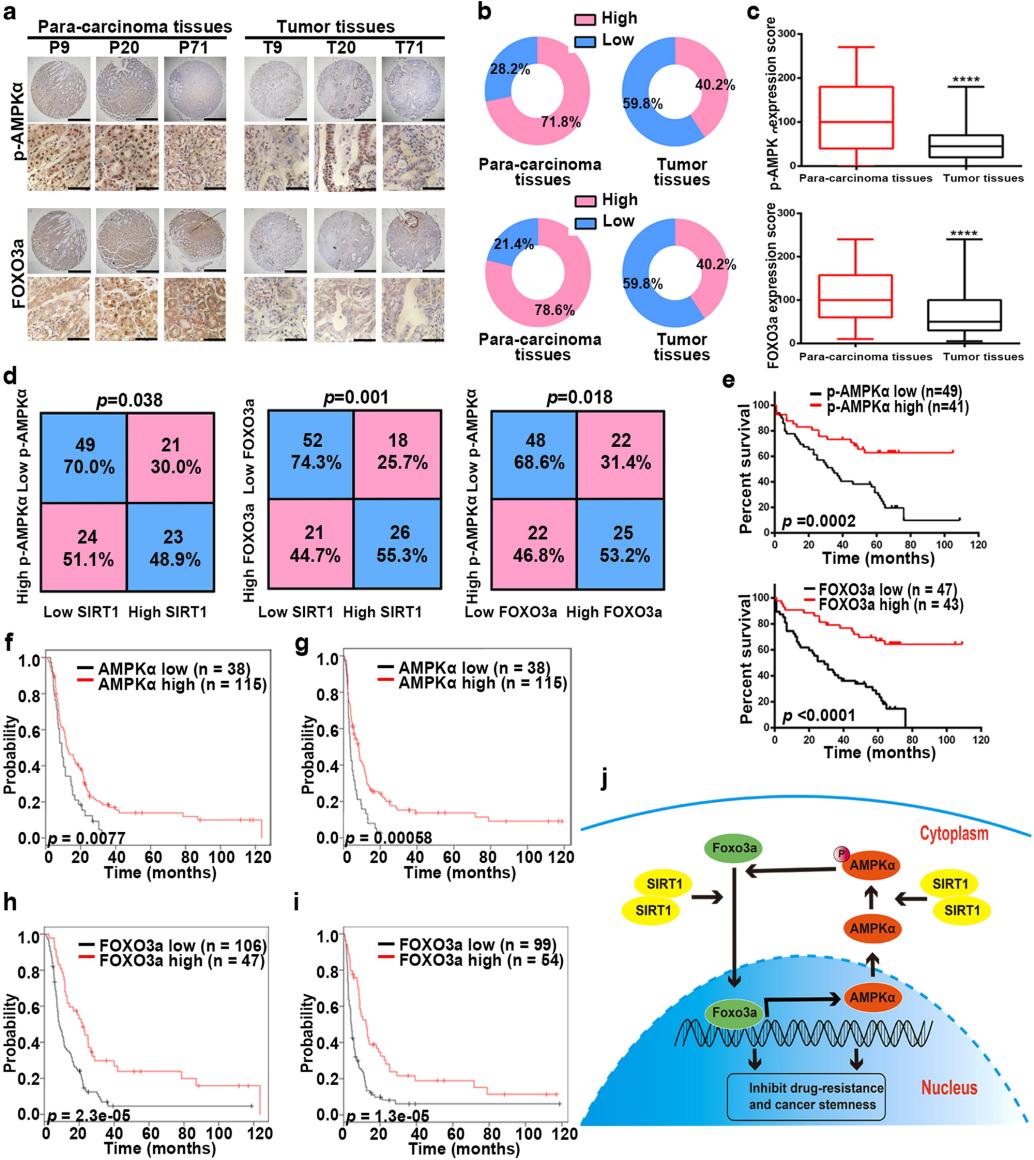
The expression levels of p-AMPKα and FOXO3a in GC patients. **a** Representative images from human GC (T) and corresponding para-carcinoma (P) tissues stained with p-AMPKα and FOXO3a (data from the tissue array). Upper panel: ×40, scale bars: 500 µm. Lower panel: ×400, scale bars: 50 µm. **b** Expression of p-AMPKα and FOXO3a in human GC (right) and corresponding para-carcinoma (left) tissues (tissue array, *n* = 117). **c** The IHC score (staining intensity × positive percentages) for p-AMPKα and FOXO3a staining in GC and corresponding para-carcinoma tissues (tissue array, *n* = 117). Data are presented as mean ± SD, **** represents for *p* < 0.0001. **d** The correlation between SIRT1, p-AMPKα, and FOXO3a expression levels in GC tissues (tissue array, *n* = 117). **e** Analysis of p-AMPKα and FOXO3a expression levels in relation to the overall survival of GC patients (tissue array, *n* = 90). **f**, **g** Analysis of AMPKα expression levels in relation to the overall survival (**f**) and first progression (**g**) of GC patients treated with a 5-FU-based regimen from the Kaplan–Meier plotter database (209799_s_at) (*n* = 153). **h**, **i** Analysis of FOXO3a expression levels in relation to the overall survival (**h**) and first progression (**i**) of GC pat**i**ents treated with a 5-FU-based regimen from the Kaplan–Meier plotter database (204132_s_at) (*n* = 153). **j** A schematic model showing the role of the SIRT1-AMPK/FOXO3 pathway in inhibition of chemoresistance and CSC properties of GC.

To determine the clinical significance of AMPK and FOXO3 in GC, we assessed their expression using the abovementioned tissue arrays. Because phosphorylated AMPKα is the active and functional form of AMPKα, p-AMPKα instead of AMPKα was examined. IHC staining showed downregulated expression of p-AMPKα and FOXO3a in GC tissues (Fig. 6a–c). In addition, the expression levels of p-AMPKα and FOXO3a in patients with low SIRT1 expression levels were significantly lower than those in patients with high SIRT1 expression levels. The correlation between expression levels of p-AMPKα and FOXO3a was also identified (Fig. 6d). Moreover, high expression levels of p-AMPKα and FOXO3a were correlated with good overall survival (Fig. 6e). Consistent with our findings, the data from the Kaplan–Meier plotter database (209799_s_at) also correlated high AMPKα expression levels with good overall survival (Supplementary Fig. S6a). Furthermore, in GC patients receiving 5-FU-based chemotherapy, high expression levels of AMPKα indicated good outcomes (Fig. 6f, g). Similar to the results of AMPKα, high expression levels of FOXO3a were correlated with a good prognosis in GC patients treated with a 5-FU-based regimen (204132_s_at) (Fig. 6h, i; Supplementary Fig. S6b–g). Moreover, using Cox regression analyses, we further confirmed that expression levels of FOXO3a (*p* < 0.001) are independent predictors of the overall survival of GC patients (Supplementary Table S1).

Taken together, our findings indicate that the SIRT1-AMPK/FOXO3 signaling pathway inhibits chemoresistance and CSC properties in GC (Fig. 6j).

## Discussion

Changes in SIRT1 expression levels are frequent molecular events in human cancers^11,19–21^. Recent data have shown low expression levels of SIRT1 in human colon cancer, lung cancer, and glioblastoma. Moreover, high expression levels of SIRT1 indicate a good prognosis in patients. Our results showed that SIRT1 expression was downregulated in human GC tissues. High expression levels of SIRT1 indicate good outcomes in GC patients. Data from the Kaplan–Meier plotter database also associate high expression levels of SIRT1 with a good prognosis in GC. Interestingly, for GC patients treated with a 5-FU-based regimen, high expression levels of SIRT1 also indicated a good prognosis. These results suggest the tumor-suppressive role of SIRT1 in GC and associate SIRT1 with the response to chemotherapy.

Currently, cisplatin- and 5-FU-based chemotherapy is the standard care for GC^2,22,23^. The consequent chemoresistance to the abovementioned treatment leads to unsatisfactory survival of GC patients. Activators of SIRT1 have been evaluated in preclinical studies and were shown to be a promising therapeutic strategy for glioblastoma and multiple myeloma^21,24^. Furthermore, in lung and pancreatic cancer, activation of SIRT1 has been shown to enhance cancer cell sensitivity to classic chemotherapy^25,26^. In this study, we provided further evidence showing that overexpression of SIRT1 improves chemotherapeutic effects in GC cells. With forced expression of SIRT1, GC cells showed a decrease in the IC50 of cisplatin and 5-FU, an increase in apoptosis upon cisplatin treatment and enhanced sensitivity to cisplatin in xenografted mice. Moreover, SIRT1 exerted inhibitory effects on CSC properties of GC. The mechanism for the suppressive role of SIRT1 in chemoresistance and CSC properties was further explored.

In *Caenorhabditis elegans*, the ability of Sir2, an NAD + -dependent deacetylase, to extend life span relies on presence of Daf-16, the FOXO transcription factor^27^. In addition to improving longevity, the SIRT1-FOXO axis was also found to play a role in alleviating insulin resistance and regulating glucose metabolism^28^. SIRT1 protects against emphysema through FOXO3-mediated reduction of cellular senescence^29^. In mouse aged kidney, the SIRT1-FOXO3 pathway improved cellular adaption to hypoxia by inducing mitochondrial autophagy^30^. In GC, FOXO3 has been shown to be expressed at low levels and exerts antitumor effects^31,32^. The results of this study demonstrated that deletion of FOXO3a reverses the effects induced by SIRT1 overexpression in GC cells. However, the activity of SIRT1 on drug resistance and CSC properties of GC cells is only partially reversed by FOXO3 knockdown, suggesting that other targets of SIRT1 also participate in this process.

AMPK, which acts as a conserved energy sensor, is the target of SIRT1 for regulation of cellular metabolism. Briefly, SIRT1 deacetylates LKB1, and then LKB1 is translocated from the nucleus to the cytoplasm, forms an active complex and activates AMPKα^25,33^. As metabolic sensors, SIRT1/AMPK signaling was shown to play important role in metabolism^33^. Recently, AMPK activated by SIRT1 is proven to act as a tumor suppressor in multiple solid tumors by inducing cell death, inhibiting cell migration, and attenuating hypoxia-induced chemoresistance^21,25,34–36^. Our data demonstrated that silencing AMPKα partially reverses inhibitory role of SIRT1 in GC cells. As AMPK was shown to be correlated with both SIRT1 and FOXO3 in STRING database, we hypothesized that both AMPK and FOXO3 participate in the inhibitory effects of SIRT1. Subsequent double knockdown of AMPKα and FOXO3a showed a complete reversal of the effects of SIRT1 on chemoresistance. These findings were further confirmed in a mouse model. Furthermore, the results in this study demonstrated that SIRT1 exerts inhibitory effects on CSC properties through AMPK and FOXO3.

Next, the potential synergistic effects between AMPK and FOXO3 were explored. It has been reported that AMPK can phosphorylate FOXO3^37^. FOXO3 phosphorylated by AMPK translocates from the cytoplasm to the nucleus with enhanced transcriptional activities^38,39^. Using immunofluorescence staining and luciferase assays, we confirmed the above effects in GC cells. In terms of FOXO3 regulation of AMPK, Tullet et al.^18^ demonstrated that FOXO directly activates the expression of AMPKγ and thus plays an important role in aging in *Caenorhabditis elegans*. AMPKβ expression is also upregulated by FOXO, but has no effect on life span. Nevertheless, no direct regulation of AMPK by FOXO3 has been identified in mammals^40,41^. Our results demonstrated that in GC cells, FOXO3a positively regulates the expression of the α-subunit of AMPK, but not the β- or γ-subunit. Direct and functional binding of FOXO3a on the promoter of AMPKα was indicated by ChIP assays and luciferase assays. In addition to expression levels, phosphorylated AMPKα, which is the active form of AMPKα, was also downregulated by FOXO3a interference. Our findings indicated that in GC cells, FOXO3 may promote AMPKα expression and activation. Thus, a positive feedback loop between AMPK and FOXO3 is identified in GC cells.

In summary, our results showed that low SIRT1 expression levels indicate a poor prognosis of GC patients. SIRT1 exerts inhibitory effects on drug responses and CSC properties of GC cells by regulating the positive feedback between AMPK and FOXO3. Similar to SIRT1, low expression levels of p-AMPKα and FOXO3a are identified in GC tissues and are related to a poor prognosis of GC patients. In addition, correlations between SIRT1, p-AMPKα, and FOXO3a were shown using human GC samples. These findings indicate the importance of the SIRT1-AMPK/FOXO3 pathway in rescuing chemoresistance and cancer stemness of GC. Thus, development of efficient strategies to activate the SIRT1-AMPK/FOXO3 pathway may eventually lead to improving the survival of GC patients.

## Materials and methods

### Cells and siRNAs

Human GC cell lines AGS, BGC-823, and SGC-7901 (Cell Resource Center, Institute of Biochemistry and Cell Biology at the Chinese Academy of Sciences, Shanghai, China) were cultured in F12 (AGS cells) or RPMI 1640 (BGC-823 and SGC-7901 cells) containing 10% FBS, 100 units/ml penicillin, and 100 µg/ml streptomycin. The cell bank routinely performs cell line authentication by short tandem repeat profiling, and all of the cell lines were passaged in our lab for no more than 6 months after receipt. Stable lentivirus-infected GC cells were constructed and maintained as previously described^42^. Mycoplasma PCR testing was performed every month (GeneCopoeia, Rockville, MD, USA). Lipofectamine 2000 (Invitrogen, Carlsbad, CA, USA) was used to transfect small interfering RNAs (siRNAs) (GenePharma, Shanghai, China). The sequences of the siRNAs are shown in Supplementary Table S2.

### MTS assay

Cells were treated with cisplatin (Sigma, St. Louis, MO, USA) (140, 28, 5.6, 1.12, 0.224, and 0 μg/ml for AGS cells; 15, 3, 0.6, 0.12, 0.024, and 0 μg/ml for BGC-823 and SGC-7901 cells) or 5-FU (Sigma) (2 500, 500, 100, 20, 4, and 0 μg/ml). Cell viability was analyzed 48 h later as previously described^42^. The IC50 value was calculated using GraphPad Prism 6 software.

### Colony-formation assay

Cells were pretreated with cisplatin and then seeded into six-well plates and incubated for 10 days. The number of colonies was counted as previously described^42^.

### Flow cytometry

For apoptosis analysis, cells were analyzed with PE Annexin V Apoptosis Detection Kit I (BD Biosciences, San Jose, CA, USA). For examination of CD44 expression, cells were stained with PE Mouse Anti-Human CD44 (#555479, BD Biosciences). Samples were examined by flow cytometry (CytoFLEX, Beckman Coulter), and the data were analyzed using CytExpert software (Beckman Coulter).

### Mammosphere assay

For the formation of mammospheres, cells were suspended in serum-free F12 (AGS cells) or RPMI 1640 (SGC-7901 cells) containing 2% B27 (Gibco, Waltham, MA, USA), 10 ng/ml FGF (Peprotech, Rocky Hill, NJ, USA), 10 ng/ml EGF (Peprotech), and 2 μg/ml Heparin (MCE, Monmouth Junction, NJ, USA). Then, the cells were seeded onto 24-well ultralow attachment plates and incubated for 7–10 days. Spheres > 50 μm were counted under a microscope.

### Soft agar colony-formation assay

Cells were suspended in complete medium with 0.3% agar (upper agar layer) and added to a 12-well plate precoated with complete medium containing 0.6% agar (lower ager layer). Complete medium was added to the surface of the upper agar layer, and was changed every 3 days. After 15–20 days, colonies > 50 µm were counted under a microscope.

### RNA extraction and quantitative real-time PCR (qRT-PCR)

The total RNA was extracted with TRIzol (Invitrogen) and converted into cDNA, which was amplified by qRT-PCR as previously described^42^. The primer sequences are shown in Supplementary Table S2.

### Western blot

The total cellular protein was isolated with RIPA Lysing Buffer (Beyotime, Shanghai, China), and the protein concentration was measured using a BCA Protein Assay Kit (Beyotime). Membranes were probed with specific primary antibodies against AMPKγ (ab32508), OCT4 (ab200834), SOX2 (ab171380) (Abcam, Cambridge, MA, USA), AMPKα (#2603), p-AMPKα (#2535), FOXO3a (#12829), Caspase-3 (#9662) and β-actin (#4967) (Cell Signaling, Danvers, MA, USA). Protein bands were visualized as previously described^42^.

### Luciferase assay

Luciferase reporter plasmids containing the promoter sequence of AMPKα, assumed FOXO3a binding sites deleted promoter sequences of AMPKα were constructed by Bioasia (Jinan, China). FHRE-Luc (#1789, Addgene) was a luciferase construct containing three copies of forkhead response elements^43^. The relative luciferase activities were measured and calculated as previously described^44^.

### Chromatin immunoprecipitation (ChIP)

ChIP assays were performed as previously described^44^ with anti-FOXO3a-ChIP Grade (ab12162, Abcam). Coprecipitated DNA served as the template for amplification of the AMPKα and AMPKγ promoters. The primer sequences are shown in Supplementary Table S2.

### Immunofluorescence staining

Cells were fixed in 4% fixative solution and permeabilized with 0.2% Triton X-100. After blocking, the cells were incubated with primary antibodies against FOXO3a (#12829, Cell Signaling), and then a fluorescent secondary antibody. Nuclei were stained with DAPI (Beyotime). Images were obtained under a microscope (Olympus, Tokyo, Japan) using CellSens Dimension software.

### Tissue arrays and immunohistochemistry (IHC) staining

Commercial tissue arrays were customized by Shanghai Outdo Biotech Company. The tissue arrays contained 117 pairs of GC tissues and corresponding para-carcinoma tissues. Clinicopathological data of all patients, survival data of 90 patients, and informed consents were provided by the manufacturer. IHC staining was performed^42^ with antibodies against SIRT1 (ab32441), p-AMPK (ab194920) and FOXO3a (ab12162) (Abcam). The staining intensity was scored as follows: no staining (score: 0), light brown (score: 1), medium brown (score: 2), and dark brown (score: 3). The expression score was calculated as follows: staining intensity score × positive percentages. To divide GC tissues into high expression-level and low expression-level groups, the proportion of positively stained cells (0–100%) was scored as follows: 0% (score: 0), ≤ 10% (score: 1), 11–50% (score: 2), 51–75% (score: 3), and >75% (score: 4). IHC grade was calculated as follows: staining intensity score × positive proportion score. Samples with an IHC grade ≥ 3 were considered high expression-level samples, while those with an IHC grade < 3 were considered low expression-level samples. Images were obtained under a microscope (Olympus) using CellSens Dimension software.

### Xenograft tumor model

The animal study was approved by the Ethical Committee of School of Basic Medical Sciences, Shandong University (ECSBMSSDU2019-2-010). Male BALB/c-nude mice (6 weeks old) were purchased from Charles River (Beijing, China). Stably lentivirus-infected SGC-7901 cells (5 × 10^6^) were subcutaneously injected into each mouse. When the tumor volumes (L × W^2^/2) reached 100 mm^3^ (regarded as day 0), the mice were injected with cisplatin or NaCl every 5 days. Measurements of the tumor volume were performed every week. On day 21, the mice were killed, and the tumor xenografts were removed, and fixed in 10% buffered formalin for TUNEL staining. The investigators were not blinded to the mice group during experiments.

For the recovery experiment, nude mice were randomly divided into five groups. In detail, group I, mice were injected with LV-C clones (regarded as LV-C + CDDP); group II, mice were injected with LV-S clones (regarded as LV-S + CDDP); group III, mice were injected with LV-S clones (regarded as LV-S + A-In + CDDP); group IV, mice were injected with LV-S clones (regarded as LV-S + F-In + CDDP) and group V, mice were injected with LV-S clones (regarded as LV-S + D-In + CDDP). When the tumor volumes reached 100 mm^3^, the mice in group III, IV, and V received inhibitors of AMPK (Compound C, Selleckchem, Houston, TX, USA, dissolved in NaCl, 20 mg/kg, i.p.), FOXO3 (AS1842856, Biochempartner, Shanghai, China, dissolved in 6% cyclodextrin, 100 mg/kg, p.o.) and both the inhibitors, respectively. The mice in groups I and II received NaCl and 6% cyclodextrin as controls. The following day was regarded as day 0. Then the mice were treated with cisplatin and killed as abovementioned.

For in vivo limiting dilution assay, stably lentivirus-infected SGC-7901 cells (2 × 10^6^, 5 × 10^5^, 2 × 10^5^, 5 × 10^4^) were subcutaneously injected into each mouse (five mice for each group). Tumor growth was assessed weekly. After 4 weeks, the mice were killed, and tumor formation was examined. The frequency of CSCs was calculated and *p*-value was evaluated using ELDA (http://bioinf.wehi.edu.au/software/elda)^45^.

### TUNEL staining

TUNEL staining for the analysis of apoptosis was performed using In Situ Cell Death Detection Kit AP and NBT/BCIP (Roche Applied Science, Basel, Switzerland). Images were obtained under a microscope (Olympus) using CellSens Dimension software.

### Statistical analysis

Comparisons between different groups were analyzed using Student’s *t* test or one-way ANOVA. Survival curves were plotted using the Kaplan–Meier method and compared using the log-rank (Mantel–Cox) test. Survival data were determined by univariate and multivariate Cox regression analyses. The correlation between SIRT1, p-AMPKα, and FOXO3a was analyzed by Spearman correlation. Statistical analysis was performed using GraphPad Prism 6 and SPSS (version 20.0). The level of statistical significance was set at *p* < 0.05.

## Supplementary information

Supplementary Table 1

Supplementary Table 2

Supplementary Figure Legends

Supplementary Figure 1

Supplementary Figure 2

Supplementary Figure 3

Supplementary Figure 4

Supplementary Figure 5

Supplementary Figure 6
