# Supplementary material for: SIRT1 inhibits chemoresistance and cancer stemness of gastric cancer by initiating an AMPK/FOXO3 positive feedback loop
Source: Cell Death Dis. 2020 Feb 12;11(2):115. doi: 10.1038/s41419-020-2308-4 (PMC7015918; doi:10.1038/s41419-020-2308-4)
Supplement: Supplementary file 1 — Supplementary Table 1 [file 41419_2020_2308_MOESM1_ESM.doc]

**Table S1.** Univariate and multivariate Cox regression analyses of overall survival.

| **Variables** | **n** | **Univariate Cox** | | | **Multivariate Cox** | | |
| --- | --- | --- | --- | --- | --- | --- | --- |
|  |  | **HR** | **95% CI** | ***p* value** | **HR** | **95% CI** | ***p* value** |
| **Gender** |  |  |  | 0.067 |  |  |  |
| Women | 23 | 1.000 |  |  |  |  |  |
| Men | 67 | 1.86 | 0.958-3.611 |  |  |  |  |
| **Age (year)** ^a^ |  |  |  | 0.36 |  |  |  |
| ≤ 65 | 46 | 1.000 |  |  |  |  |  |
| > 65 | 44 | 1.284 | 0.752-2.192 |  |  |  |  |
| **Tumor size (cm^3^)** ^a,b^ |  |  |  | 0.111 |  |  |  |
| ≤ 52 | 45 | 1.000 |  |  |  |  |  |
| > 52 | 44 | 1.554 | 0.903-2.674 |  |  |  |  |
| **Tumor infiltration** |  |  |  | 0.03^c^ |  |  | 0.716 |
| T1 | 13 | 1.000 |  |  | 1.000 |  |  |
| T2/T3 | 11 | 2.173 | 0.537-8.785 |  | 0.692 | 0.053-9.128 |  |
| T4 | 66 | 4.059 | 1.258-13.099 |  | 1.304 | 0.060-28.187 |  |
| **Local lymph node metastasis** |  |  |  | < 0.001^c^ |  |  | 0.022^c^ |
| 0 | 30 | 1.000 |  |  | 1.000 |  |  |
| 1 | 11 | 2.415 | 0.837-6.966 |  | 1.819 | 0.287-11.516 |  |
| 2 | 20 | 3.676 | 1.554-8.7 |  | 2.536 | 0.455-14.138 |  |
| 3 | 29 | 6.483 | 2.891-14.54 |  | 5.716 | 0.952-34.328 |  |
| **Clinical stage**^d^ |  |  |  | 0.001^c^ |  |  | 0.354 |
| I | 16 | 1.000 |  |  | 1.000 |  |  |
| II | 18 | 1.491 | 0.436-5.098 |  | 0.17 | 0.012-2.344 |  |
| III/IV | 56 | 4.66 | 1.664-13.049 |  | 0.186 | 0.005-6.546 |  |
| **Grade** |  |  |  | 0.044^c^ |  |  | 0.077 |
| I | 13 | 1.000 |  |  | 1.000 |  |  |
| II | 14 | 6.429 | 1.383-29.879 |  | 12.863 | 1.191-138.956 |  |
| III | 63 | 5.946 | 1.438-24.588 |  | 7.132 | 0.755-67.360 |  |
| **SIRT1**  **expression levels** |  |  |  | < 0.001^c^ |  |  | 0.013^c^ |
| Low | 49 | 1.000 |  |  | 1.000 |  |  |
| High | 41 | 0.284 | 0.153-0.527 |  | 0.407 | 0.201-0.825 |  |
| **p-AMPKα**  **expression** **levels** |  |  |  | < 0.001^c^ |  |  | 0.216 |
| Low | 49 | 1.000 |  |  | 1.000 |  |  |
| High | 41 | 0.339 | 0.186-0.618 |  | 0.663 | 0.345-1.272 |  |
| **FOXO3a**  **expression levels** |  |  |  | < 0.001^c^ |  |  | < 0.001^c^ |
| Low | 47 | 1.000 |  |  | 1.000 |  |  |
| High | 43 | 0.252 | 0.137-0.461 |  | 0.280 | 0.141-0.558 |  |

n, Numbers of cases in each group.

HR, hazard ratio.

CI, confidence interval.

^a^ Median.

^b^ The data of tumor size in one patient is not available.

^c^ Statistically significant (*p* < 0.05).

^d^ AJCC Cancer Stage Manual, 7^th^ Edition (2010).
