# Supplementary material for: SIRT1 inhibits chemoresistance and cancer stemness of gastric cancer by initiating an AMPK/FOXO3 positive feedback loop
Source: Cell Death Dis. 2020 Feb 12;11(2):115. doi: 10.1038/s41419-020-2308-4 (PMC7015918; doi:10.1038/s41419-020-2308-4)
Supplement: Supplementary file 2 — Supplementary Table 2 [file 41419_2020_2308_MOESM2_ESM.doc]

**Table S2.** Sequences of siRNAs and primers.

|  | **Gene** | **Sequence (5’ → 3’)** |
| --- | --- | --- |
| **siRNA** | *FOXO3a*-1 | CCUCAUCUCCACACAGAAUTT |
|  | *FOXO3a*-2 | GCUCACUUCGGACUCACUUTT |
|  | *AMPKa*-1 | GAGGAGAGCUAUUUGAUUATT |
|  | *AMPKa*-2 | GCGUGUACGAAGGAAGAAUTT |
|  | *SIRT1* | CCAUCUCUCUGUCACAAAUTT |
|  | *Negative control* | UUCUCCGAACGUGUCACGUTT |
| **Primers** | *SIRT1* | TGGCAAAGGAGCAGATTAGTAGG  CTGCCACAAGAACTAGAGGATAAGA |
|  | *FOXO3a* | GGCAAAGCAGACCCTCAAAC  TGTCCACTTGCTGAGAGCAG |
|  | *AMPKα* | GGAGCCTTGATGTGGTAGGA  CATCCAGCCTTCCATTCTTACAG |
|  | *AMPKβ* | TGGAATCGAGATAGCCTCGC  TGATGGGGGTCTGCCCA |
|  | *AMPKγ* | GCGTGTGGTGGACATCTACT  CAAGTCGGTGAACCTCTGCT |
|  | *OCT4* | AGCGAACCAGTATCGAGAAC  TTACAGAACCACACTCGGAC |
|  | *SOX2* | GACAGTTACGCGCACATGAA  TAGGTCTGCGAGCTGGTCAT |
|  | *NANOG* | CCCCAGCCTTTACTCTTCCTA  CCAGGTTGAATTGTTCCAGGTC |
|  | *c-MYC* | GTAGTGGAAAACCAGCAGCC  AGAAATACGGCTGCACCGAG |
|  | *CD44* | TCCAACACCTCCCAGTATGACA  GGCAGGTCTGTGACTGATGTACA |
|  | *β-actin* | TTGCCGACAGGATGCAGAA  GCCGATCCACACGGAGTACT |
|  | *AMPKα* promoter  (FOXO3a binding site-1) | GCTTTACAGAGATGCCTTGTG  TGAGGACTGCACATTGTTTGAAG |
|  | *AMPKα* promoter  (FOXO3a binding site-2) | ATCAGAGGAGTGGAATTGAGTAAC  TGGACTGAGAGAGAACAAGGCT |
|  | *AMPKα* promoter  (FOXO3a binding site-3) | GGTTGATGTAACCGTAAGCCT  CAAGAGGGGGATGATACGCAG |
|  | *AMPKγ* promoter  (FOXO3a binding site) | GAAAAGGGGATGGGGCAGAC  ACCTTGTAGCTTGGGTCTGAG |
