# Supplementary material for: SIRT1 inhibits chemoresistance and cancer stemness of gastric cancer by initiating an AMPK/FOXO3 positive feedback loop
Source: Cell Death Dis. 2020 Feb 12;11(2):115. doi: 10.1038/s41419-020-2308-4 (PMC7015918; doi:10.1038/s41419-020-2308-4)
Supplement: Supplementary file 3 — Supplementary Figure Legends [file 41419_2020_2308_MOESM3_ESM.doc]

**Supplementary figure legends**

**Fig. S1 SIRT1 expression levels and the prognosis of GC patients.** Analysis of SIRT1 expression levels in relation to the overall survival (**a,** n = 876) and first progression (**b,** n= 641) of GC patients from the Kaplan-Meier Plotter database (218878_s_at)

**Fig. S2 SIRT1 and chemoresistance of GC cells.** **a** The IC50 of 5-FU was calculated by MTS assays. The mean values of the IC50 are shown (n = 3). **b-c** Colony formation assays were performed to analyze cell proliferation upon cisplatin treatment (1.5 µg/ml, 4 h). Representative images are shown in (**b**). Data are presented as mean ± SD (n = 3).** represents *p* < 0.01, *** represents *p* < 0.001

**Fig. S3 SIRT1 and cancer stemness of GC cells. a-b** Soft agar colony formation assays were performed to evaluate the CSC properties of GC cells. Representative images are shown in (**a**). Data are presented as mean ± SD (n = 3). **c-h** Quantitative real-time PCR was performed to analyze the mRNA expression levels of *CD44* and four core transcription factors for maintaining stemness, including *OCT4*, *SOX2*, *NANOG* and *c-MYC*. Stably SIRT1-overexpressing and SIRT1-silenced GC cells were harvested, and the mRNA expression levels of *CD44* (**c** and **d**) and the abovementioned four transcription factors (**e** and **f**) were analyzed. The results from GC cells (regarded as primary) and mammospheres obtained from GC cells are shown in (**g**). The results from GC cells treated with cisplatin (CDDP, 10 µg/ml for AGS cells and 1.5 µg/ml for SGC-7901 cells, 48 h) or NaCl (Control) are shown in (**h**). Data are presented as mean ± SD (n = 3). *** represents *p* < 0.001. **i**-**j** Western blot was performed to analyze the protein levels of OCT4 and SOX2. The results from GC cells treated with cisplatin (CDDP, 10 µg/ml for AGS cells and 1.5 µg/ml for SGC-7901 cells, 48 h) or NaCl are shown in (**i**). The results from stable lentivirus-infected GC cells are shown in (**j**)

**Fig. S4 SIRT1 activates AMPK and FOXO3 in GC cells.** **a** Pathway enrichment analysis of SIRT1, core stemness factors and their associated proteins by STRING database. **b** Transcriptional activity analysis of FOXO3. GC cells were transfected with small interfering RNA targeting SIRT1 (Si) or the negative control (Ni). Data are presented as mean ± SD (n = 3). *** represents *p* < 0.001. **c** Pathway enrichment analysis of SIRT1, FOXO3 and their associated proteins by STRING database. **d** Western blot was performed to analyze the expression levels of AMPKα and p-AMPKα after treating GC cells with a SIRT1 activator (SRT1720, indicated concentration for 24 h) or a SIRT1 inhibitor (EX527, indicated concentration for 12 h)

**Fig. S5 Efficient knockdown of AMPKα and FOXO3a by siRNAs.** **a** and **c** Real-time PCR was performed to analyze the mRNA expression levels of *FOXO3a* (**a**) and *AMPKα* (**c**). Data are presented as mean ± SD (n = 3). *** represents *p* < 0.001. **b** and **d** Western blot was used to analyze the protein expression levels of FOXO3a (**b**) and AMPKα (**d**). GC cells were transfected with small interfering RNAs targeting FOXO3a (Fi) or AMPKα (Ai). Ni represents the negative control

**Fig. S6 Low expression levels of AMPKα and FOXO3a are related to a poor prognosis of GC patients (data from the Kaplan-Meier Plotter database).** **a** Analysis of AMPKα expression levels in relation to the overall survival of GC patients (209799_s_at). **b-c** Analysis of FOXO3a expression levels (217399_s_at) in relation to the overall survival (**b**) and first progression (**c**) of GC patients treated with a 5-FU-based regimen. **d-e** Analysis of FOXO3a expression levels (210655_s_at) in relation to the overall survival (**d**) and first progression (**e**) of GC patients treated with a 5-FU-based regimen. **f-g** Analysis of FOXO3a expression levels (204131_s_at) in relation to the overall survival (**f**) and first progression (**g**) of GC patients treated with a 5-FU-based regimen
