# Supplementary figures and images for: SIRT1 inhibits chemoresistance and cancer stemness of gastric cancer by initiating an AMPK/FOXO3 positive feedback loop
Source: Cell Death Dis. 2020 Feb 12;11(2):115. doi: 10.1038/s41419-020-2308-4 (PMC7015918; doi:10.1038/s41419-020-2308-4)

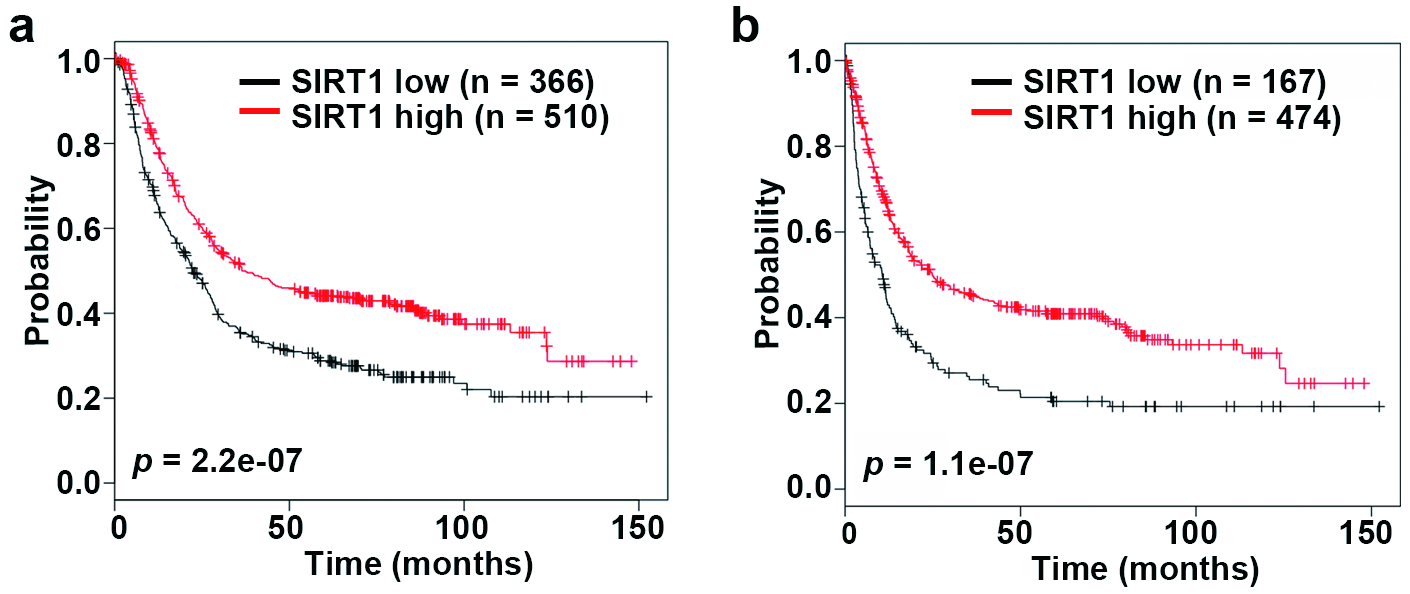

Supplement: Supplementary file 4 — Supplementary Figure 1 [file 41419_2020_2308_MOESM4_ESM.tif]

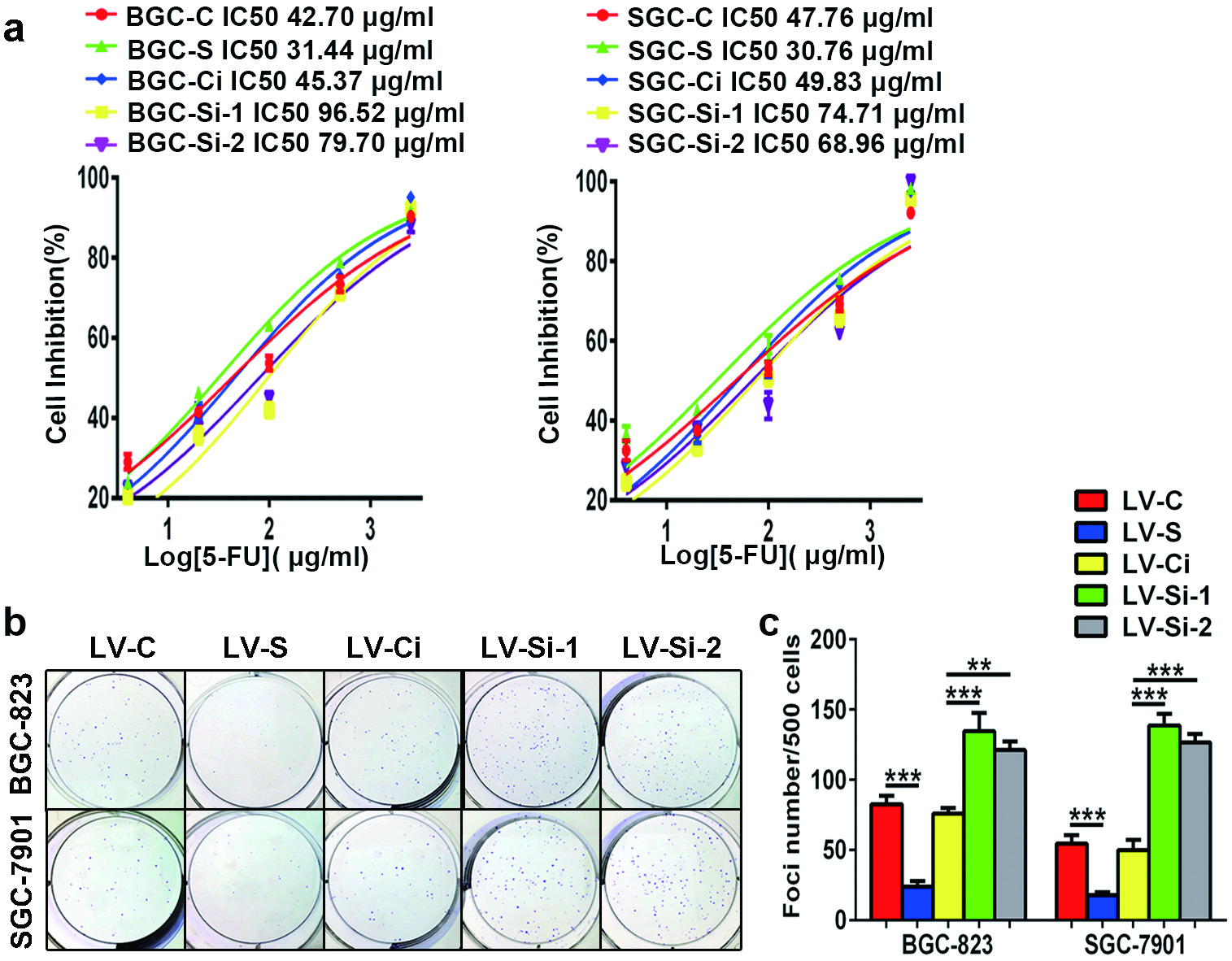

Supplement: Supplementary file 5 — Supplementary Figure 2 [file 41419_2020_2308_MOESM5_ESM.tif]

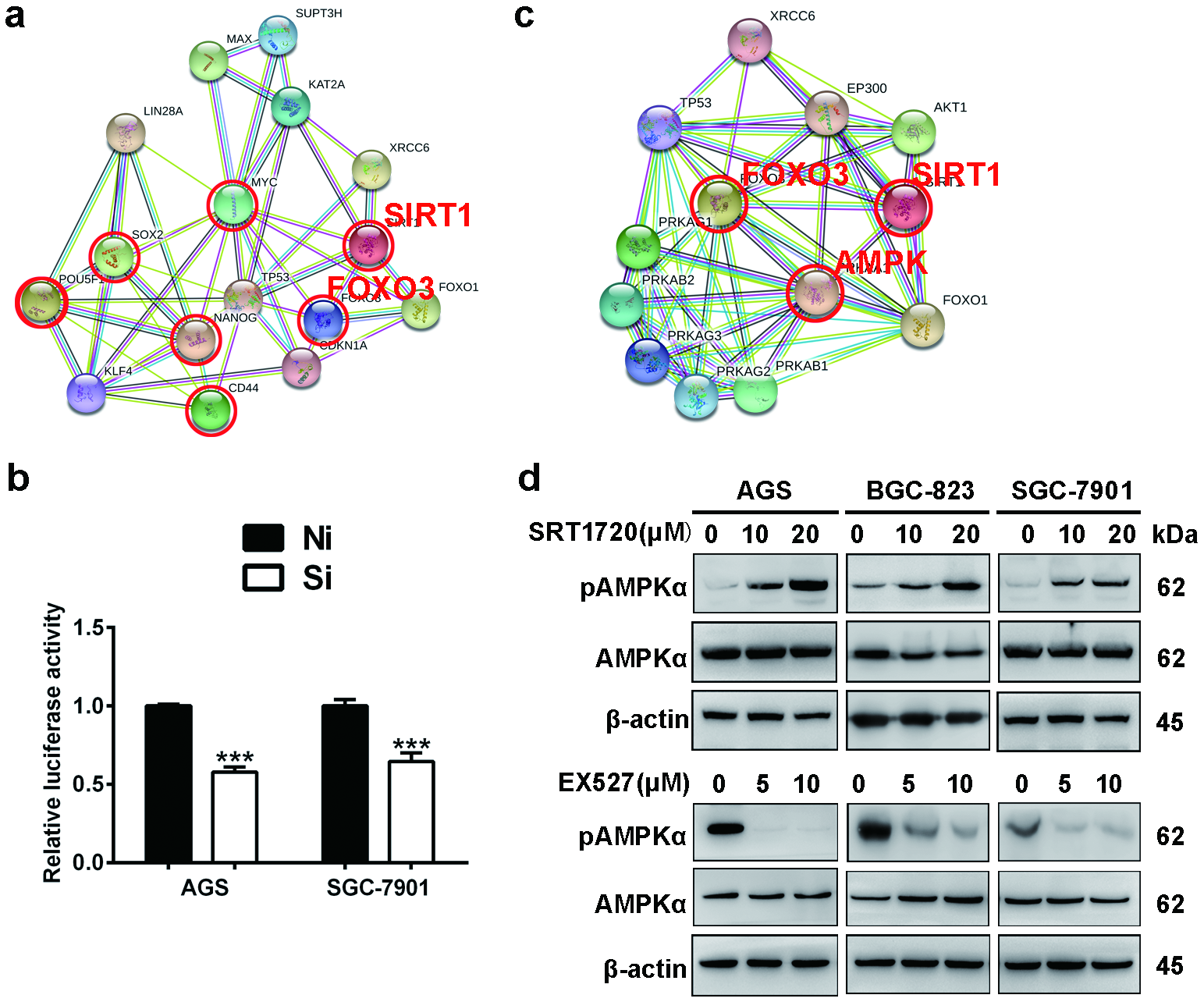

Supplement: Supplementary file 7 — Supplementary Figure 4 [file 41419_2020_2308_MOESM7_ESM.tif]

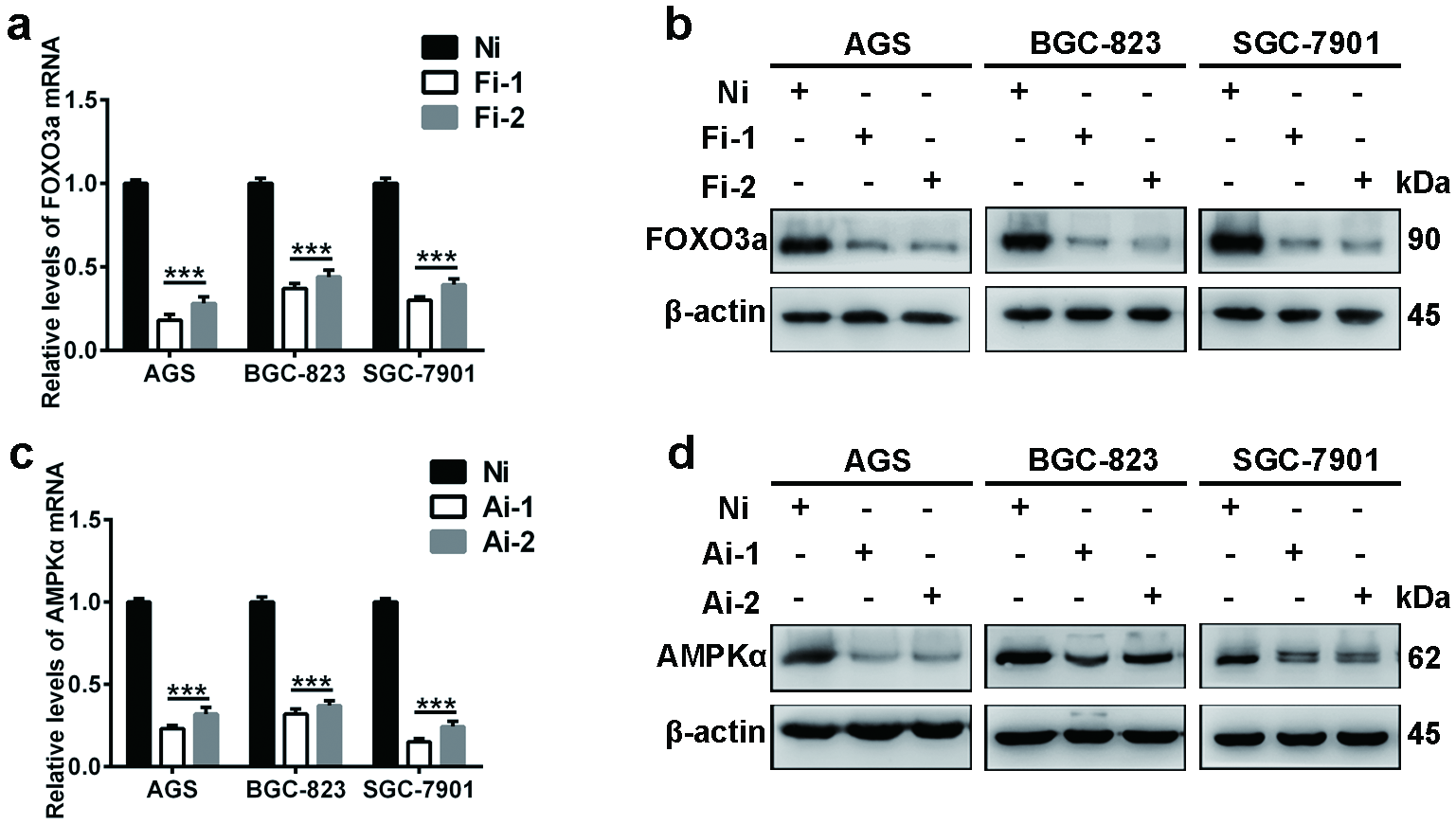

Supplement: Supplementary file 8 — Supplementary Figure 5 [file 41419_2020_2308_MOESM8_ESM.tif]

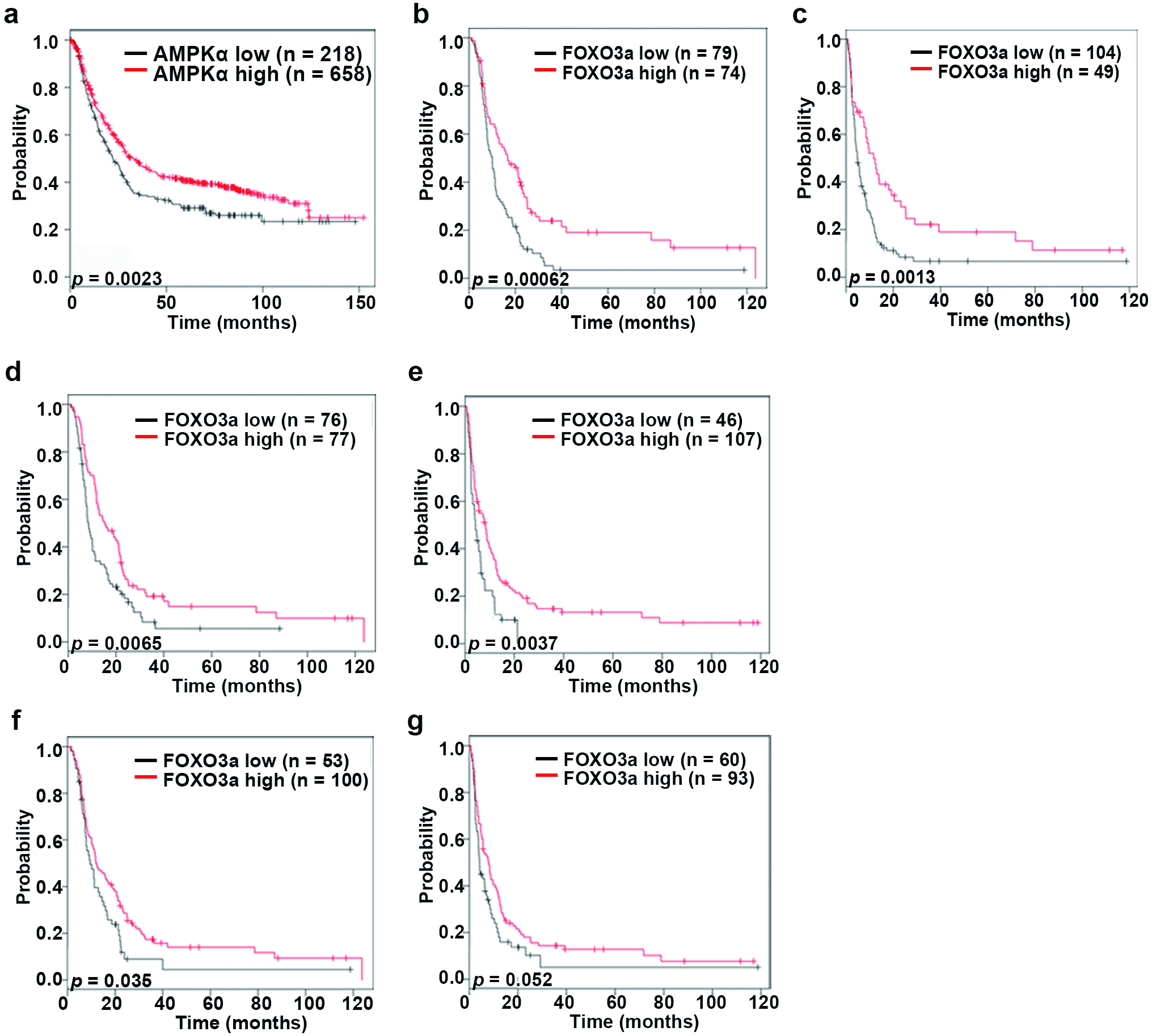

Supplement: Supplementary file 9 — Supplementary Figure 6 [file 41419_2020_2308_MOESM9_ESM.tif]
